# Nurr1 dependent regulation of pro-inflammatory mediators in immortalised synovial fibroblasts

**DOI:** 10.1186/1476-9255-2-15

**Published:** 2005-11-25

**Authors:** Mark R Davies, Christine J Harding, Stephanie Raines, Kurt Tolley, Andrew E Parker, Mark Downey-Jones, Maurice RC Needham

**Affiliations:** 1 Respiratory and Inflammation Research Department, AstraZeneca, Mereside, Alderley Park, Macclesfield, Cheshire, SK10 4TG, UK

## Abstract

**Background:**

Nurr1 is an orphan member of the nuclear receptor superfamily; these orphan receptors are a group for which a ligand has yet to be identified. Nurr1 has been shown to regulate the expression of a small number of genes as a monomeric, constitutively active receptor. These Nurr1 regulated genes are primarily associated with dopamine cell maturation and survival. However, previous reports have shown an increased expression of Nurr1 in the synovium of patients with rheumatoid arthritis (RA) suggesting a pro-inflammatory role for Nurr1 in RA. In this study we investigate the potential pro-inflammatory role of Nurr1 by monitoring Nurr1 dependent gene expression in an immortalised synoviocyte cell line, K4IM.

**Methods:**

We overexpressed the wild type and a dominant negative form of the orphan nuclear receptor Nurr1, in a model synoviocyte cell line. Using the Affymetrix HG-U133 Genechips we demonstrate the effects on the transcriptome by the receptor. Further evidence of gene expression change was demonstrated using quantitative RT-PCR and ELISA analysis.

**Results:**

We show that Nurr1 regulates transcription of a small number of genes for pro-inflammatory modulators of which the most significant is interleukin-8 (IL-8). We also demonstrate increased synthesis and secretion of IL-8 further supporting a role for Nurr1 in inflammatory signalling pathways.

**Conclusion:**

Using microarray analysis we show that elevated levels of Nurr1 leads to increased gene expression of pro-inflammatory genes: IL-8, Amphiregulin and Kit ligand in a model cell line. This data provides further evidence for an additional role for Nurr1 in inflammation and may play a role in the pathogenesis of rheumatoid arthritis.

## Background

Nuclear receptors can generally be described as ligand activated transcription factors that form a large superfamily of proteins. In humans 48 such receptors have been identified [1] and are involved with an extensive number of cellular processes throughout development and adult physiology [2]. Nuclear receptors are activated through binding by a diverse range of natural and synthetically produced ligand molecules including hormones, fatty acids and antibiotics. In addition to the receptors known to bind ligand, a group of nuclear receptors exist for which a ligand has not been identified; these are termed the orphan nuclear receptors. Among this group of orphans is Nurr1 (NR4A2), a member of the NR4 group of orphan nuclear receptors together with Nur77 (NR4A1) and NOR-1 (NR4A3) [3]. This family can bind as monomers to DNA response elements in the promoters of genes and activate transcription in the absence of ligand [4]. Interestingly, this family of receptors are also capable of binding as a heterodimer with the 9-*cis*-retinoic acid receptor, RXR [5] or as a heterodimer with other Nur-family members [6]. RXR as a heterodimer with Nurr1 remains active, suggesting that regulation can be modified by the use of specific rexinoids to enhance the response of these receptors to growth factors and therefore this could provide a novel therapeutic avenue for treatment of Nurr1 regulated disease.

The structure of Nurr1 has recently be solved highlighting differences between Nurr1 and the known liganded nuclear receptors [7]. Based upon homology modelling, the region in Nurr1 that would normally contain the ligand-binding pocket has been shown to be substantially different from other nuclear receptors suggesting that there is insufficient space in the putative ligand binding pocket to accommodate a ligand. This may explain the observations that Nurr1 is able to activate transcription in a ligand independent manner and why no ligand has yet been reported for Nurr1.

In contrast to the majority of nuclear receptors, Nurr1 is encoded by an immediate early gene that is rapidly induced in cells in response to external stimuli such as cytokines. Nurr1 has been implicated in a number of human diseases including Parkinson's disease [8,9], schizophrenia [10], alcohol dependence [11] and rheumatoid arthritis [12]. In accordance with the nature of these diseases, the expression of Nurr1 is observed in the developing and adult CNS and within the inflamed synovium of the rheumatic joint [12-14].

A number of genes have been demonstrated to be regulated by Nurr1; many of these are involved with the development and maintenance of midbrain dopaminergic neurons. These include tyrosine hydroxylase [4], Ret tyrosine kinase [15] and the dopamine transporter (SLC6A3) [16]. The Nur-family members have also been shown to play a pivotal role in regulating expression of CRH and pro-opiomelanocortin (POMC) within the hypothalamic-pituitary-adrenal (HPA) axis [6,17-19]. These studies, carried out in mouse pituitary cells, also demonstrate that CRH is capable of causing increased expression of Nurr1, suggesting the presence of a positive feedback loop. More recently, studies in synovial tissue taken from the rheumatoid joint have shown Nurr1 to be highly expressed. In addition it has been demonstrated that inflammatory cytokines are capable of increasing Nurr1 expression through NF-κB and CREB dependent signalling and this elevation in Nurr1 leads to increased transcription of CRH [12,20]. Whilst in the HPA axis, these close Nur-family members are capable of sharing overlapping roles, in primary synoviocytes, treatment with various cytokines shows predominantly Nurr1 upregulation [20]. Therefore in rheumatic synovium it is proposed that Nurr1 is acting to exacerbate the inflammatory response by driving a Nurr1-CRH positive feedback loop, indicating its potential as a target for possible therapeutic intervention.

The purpose of this study was to further investigate the role of Nurr1 in regulating inflammatory processes in synovial cells, and by using transcript profiling to identify Nurr1 regulated genes in synoviocytes, which may be playing a role in the pathology of rheumatoid arthritis.

## Methods

### Plasmid expression constructs

A reporter gene construct was generated by ligating oligonucleotides representing 3 tandem repeated consensus Nurr1 binding sites (NurRE), into the SpeI/AflII site of the SW-gal construct as previously described [21], to generate the construct: pNurRE3gal. The sense strand oligonucleotides (5'-3') for the insert were as follows, 3XNurRE: ctagtgtgacctttattctcaaaggtcagtgacctttattctcaaaggtcagtgacctttattctcaaaggtcac. Construction of the control plasmid pCMV/hGH has been previously described [22]. Dominant negative constructs were produced by fusing the DNA binding domain of Nurr1 (aa94–365) and the Drosophila *engrailed *domain (aa2–298) into the pcDNA3.1 expression vector to give pcDNA3.1-Nurr1-DN. The Nurr1 wild type gene was PCR amplified from whole brain RNA and cloned into the pcDNA3.1-V5-HIS vector and the DNA sequence verified.

### Cell culture

K4IM cells (a generous gift from E. Murphy) were cultured in RPMI-1640 media supplemented with 10% (v/v) foetal calf serum (FCS), 2 mM glutamine and 50 μg/ml penicillin-streptomycin (Gibco BRL). HeLa cells were cultured in DMEM media supplemented with 10% (v/v) foetal calf serum (FCS), 2 mM glutamine and 50 μg/ml penicillin-streptomycin (Gibco BRL). For reporter gene assays, HeLa cells were cultured in phenol red-free DMEM supplemented with 0.5% (v/v) FCS, 2 mM glutamine and 50 μg/ml penicillin-streptomycin.

### Reporter gene assays

24 hrs prior to transfection, 2.2 × 10^6 ^HeLa cells were seeded in a 9 cm^2 ^dish, in full growth medium. Cells were transfected by calcium phosphate precipitation with a total of 20–25 μg DNA per 1 ml of precipitate as previously described [23]. Generally, 10–15 μg of reporter construct was co-transfected with 2–5 μg of Nurr1 or empty vector DNA (pcDNA3.1) and including 0.5 μg CMV/hGH plasmid for data normalisation. 6 hrs post-transfection the cells were exposed to osmotic shock (15% glycerol in DMEM for 90 seconds) and the media replaced with phenol-red free DMEM supplemented with 0.5% FCS (assay medium). Cells were harvested 16–20 hrs later by incubating the cells with trypsin-EDTA (phenol red free) for 3 min at 37°C. Cells were counted, seeded into 96 well microtitre plates at 10^5 ^cells/well in 100 μl of assay medium and incubated for a further 16–20 hrs. β-galactosidase activity was measured using the spectrophotometric substrate, CPRG (Boehringer) as previously described [21]. Briefly, 100 μl of a cocktail containing 7 μl 50 mM CPRG, 7 μl Z buffer pH7 (600 mM Na_2_HPO_4_; 400 mM NaH_2_PO_4_; 100 mM KCl; 10 mM MgSO_4_; 500 mM β-mercaptoethanol), 1 μl 20% SDS and 85 μl dH_2_O water was added directly to each well and the plates incubated at 37°C before an OD 570 nm measurement was taken. Incubation times varied depending on the individual assays but were typically 30 min–3 hrs. Secreted growth hormone from the cell supernatants was measured using a 2-antibody sandwich ELISA as previously described [22]. β-galactosidase values were normalised using the hGH values.

### Determination of IL-8 protein levels

IL-8 was quantified using ELISA (R&D systems – D8050). K4IM cells were resuspended in Amaxa solution "R" to a concentration of 0.4 × 10^6 ^cells per 100 μl. A total of 2 μg of DNA was transfected using the Amaxa Nucleofector following the manufacturer's protocol (A-23). Media was collected 48 hr post nucleofection and used undiluted in duplicate according to the manufacturer's instructions.

### Quantitative RT-PCR

TaqMan real-time quantitative polymerase chain reaction (PCR) assay was performed using an ABI Prism 7700 Sequence Detection System, according to the manufacturer's protocol (Applied Biosystems), sequences for primers and probes can be found in Table 1. Additionally, primers and probes for IL-8 were obtained as a pre-formulated 20× mix from Applied Biosystems. Amplification of GAPDH (primer/probe mix 4310884E) was performed to standardize the quantification of target RNA, allowing relative quantitation using the ABI Prism 7700 SDS v1.9 software. Briefly, 25 ng of a mixture of brain, placenta and testis total RNA (Ambion 7962, 7950 & 7972) and subsequent 5-fold serial dilutions down to 1/3125 of neat were amplified in triplicate for both GAPDH and each target gene to produce a standard curve. RNA was extracted from cells using TRIzol reagent (Gibco-BRL) according to the manufacturer's guidelines. Total RNA was analysed and quantified using the Agilent Bioanalyser 2100 with the RNA Nano6000 chip. For the purpose of Taqman and RT-PCR analysis, the RNA was DNase treated using the DNase-Away kit (Ambion) according to manufacturer's protocol. 5 μl of RNA at a concentration of 5 ng/μl was dispensed in triplicate into optical 96-well plates for Taqman RT-PCR. Each sample was supplemented with both respective forward, reverse primer and fluorescent labelled probe in a total reaction volume of 25 μl using Taqman Quantitect Probe Master-Mix and RT enzyme mix (Qiagen – 204443). Each target probe was amplified in a separate 96-well plate. All samples were incubated for an initial reverse transcription reaction at 50°C for 30 minutes and then at 95°C for 15 minutes, followed by 40 cycles at 95°C for 15 seconds and 60°C for 1 minute.

**Table 1:** Sequences for Taqman RT-PCR. Sequences for primers and probes were designed using Primer Express (Applied Biosystems).

**Gene**	**Size of product**	**Primer**	**Sequence (5'-3')**
Nurr1	77 bp	Forward	TGTGTTCAGGCGCAGTATGG
		Reverse	TCCCGAAGAGTGGTAACTGTAGC
		Probe	CCTCGCCTCAAGGAGCCAGCC
AREG	70 bp	Forward	ACTCGGCTCAGGCCATTATG
		Reverse	AAAATGGTTCACGCTTCCCA
		Probe	TGCTGGATTGGACCTCAATGACACCTACT
KITLG	75 bp	Forward	TGGTGGCAAATCTTCCAAAAG
		Reverse	CAATGACTTGGCAAAACATCCA
		Probe	CATGATAACCCTCAAATATGTCCCCGGG

### DNA microarray

Nucleofection was used to transfect the following three expression constructs into the K41M cell line: 1. pcDNA3.1 blank vector (control); 2. pcDNA3.1-Nurr1-WT (Nurr1 WT); 3. pcDNA3.1-Nurr1^194–365^EnR^2–298 ^dominant negative (DN) co-transfected with pcDNA3.1-Nurr1-WT (Nurr1 DN/Nurr1 WT). Each transfection was carried out in triplicate, thus generating 9 samples from which RNA was extracted. Total RNA was extracted from each sample using RNeasy kit (Qiagen – 74104). RNA integrity and yield were analysed and quantified using the Agilent Bioanalyser 2100 with the RNA Nano6000 chip prior to synthesis of cRNA probes. Preparation of cRNA, hybridization and scanning of the HG-U133 GeneChip oligonucleotide arrays were performed according to the manufacturer's protocol (Affymetrix, Santa Clara, CA). GeneChip images were quantified and gene expression values were calculated by Affymetrix Microarray suite version 5.0 (Mas 5.0, Affymetrix). For statistical analysis a one-way analysis of variance (ANOVA) was used to compare Nurr1 WT to control samples. Prior to the test, probesets that were absent and/or had an expression signal less than 200 in all samples were removed. Probesets that had P-values less than 0.01 were considered statistically significant. A similar one-way ANOVA analysis across each probeset was used to compare Nurr1 DN to control samples. Candidate genes were retained if they showed greater than 1.5-fold upregulation, (p < 0.01) between the control and Nurr1 WT samples and yet no significant regulation (p < 0.01) between the control and Nurr1 DN samples. These remaining genes were then manually chosen for genes exhibiting an ideal profile (being changed with Nurr1 and returned towards basal activity with Nurr D/N).

## Results

### Dominant negative Nurr1 blocks Nurr1 induced reporter gene expression

**Figure 1:**
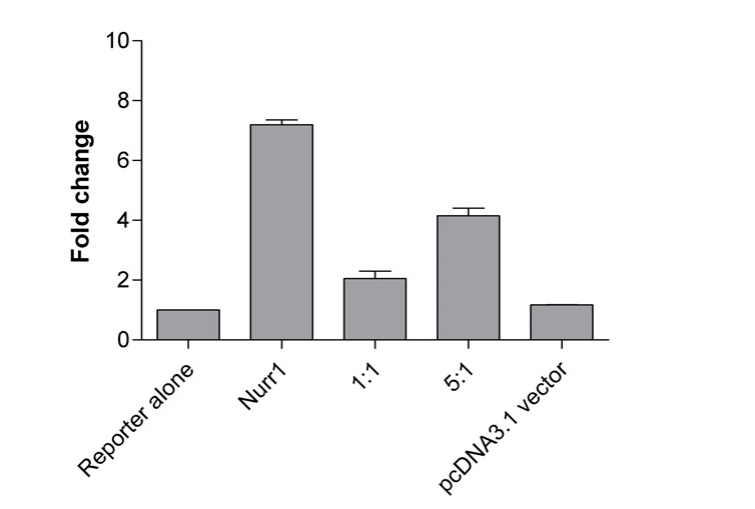
**Coexpression of a dominant negative-Nurr1 receptor attenuates the transactivation activity of Nurr1-wild type in a β-Gal reporter assay**. HeLa cells coexpressing pCDNA3.1-Nurr1-WT and pcDNA3.1-Nurr1-DN in varying ratios demonstrates strong antagonist effects of dominant negative Nurr1 construct on Nurr1 transcriptional activity (pNurRE3gal), values normalised to cotransfected hGH, using an hGH sandwich ELISA assay. Values expressed represent the mean fold change ± SEM, compared to the reporter alone.

To demonstrate the transcriptional activity of Nurr1 and its subsequent attenuation with dominant negative constructs, we used a reporter gene assay to show induction of the *LacZ *gene under the control of NurRE's. In HeLa cells transfected with the wildtype Nurr1 expression vector, activation of NurRE driven reporter gene constructs was observed. This is consistent with previous reports showing that in the absence of a ligand this family of receptors are constitutively active when assayed using transfected cell-lines [24,25]. In order to assess the specific role of Nurr1 on gene expression in K4IM cells we used a dominant negative Nurr1, which contained the DNA binding domain of the Nurr1 (2–298) fused to the *Drosophila *engrailed repressor domain. This type of dominant negative has been used extensively to study the role of transcription factor knockout phenotypes [26,27]. Transfection of K4IM cells with wildtype Nurr1 expression constructs causes induction of expression of the reporter gene whilst co-transfection of the dominant negative with the wild-type Nurr1 attenuates this effect at a range of concentrations (Figure 1). This dominant negative Nurr1 activity was therefore used to assess the specific role of Nurr1 activity in K4IM cells.

### Analysis of Nurr1 mediated gene expression in K4IM cells

The synovial fibroblast cell line K4IM was chosen as a model cell line to explore the role of Nurr1 in pro-inflammatory signalling pathways relevant to the arthritic joint [12,28]. Cells were transfected with Nurr1 constructs and RNA extracted from cells 16 hours post nucleofection. This was used to prepare cRNA probes for hybridisation to HG-U133 gene chip arrays. Quality control assessment of the Genechip arrays identified that scaling factors were less than 3 fold apart, ratios of 5' versus 3' probe sets for GAPDH and β-actin were close to 1 and background and noise levels were acceptable. Genes were considered to be Nurr1 dependent only if they exhibited a greater than 2 fold change (Nurr1-WT transfected cells compared to vector control) and only if their expression was attenuated by co-transfection with the Nurr1 DN expression construct (see Table 2). Only three genes were identified with this profile Interleukin-8 (IL-8), Amphiregulin (AREG) and Kit ligand (KITLG). The greatest change was seen with IL-8, which was induced 5-fold (p = 10^-4^).

**Table 2:** Differentially expressed genes following Nurr1 overexpression. K4IM cells were transfected in triplicate using the Amaxa Nucleofector system for each of the 3 conditions: 1. 5 μg pcDNA3.1 blank vector (control); 2. 2.5 μg pcDNA3.1-Nurr1-WT (Nurr1 WT) and 2.5 μg pCDNA3.1 blank vector; 3. 2.5 μg pcDNA3.1-Nurr1-DN dominant negative (DN) co-transfected with 2.5 μg pCDNA3.1-Nurr1-WT (Nurr1 DN/Nurr1 WT). Cells were cultured for 16 hours prior to RNA extraction. Genes were identified from the Affymetrix U133A chip showing significant change between blank vector transfected synoviocytes and Nurr1 transfected K4IM cells (>2 fold) and for genes not significantly changed between blank vector and Nurr1 D/N transfected cells (with a p-value of < 0.01). In each case genes were manually selected that showed subsequent return to basal levels with dominant negative cotransfection.

**Official HUGO Symbol**	**Description**	**RefSeqN Id**	**Fold change**	**p-value**
IL8	Interleukin 8	NM_000584	5.04	0.00047
AREG	Amphiregulin (schwannoma-derived growth factor)	NM_001657	2.80	0.00037
KITLG	KIT ligand	NM_000899	2.23	0.00390

### Nurr1 dependent regulation of pro-inflammatory genes

**Figure 2:**
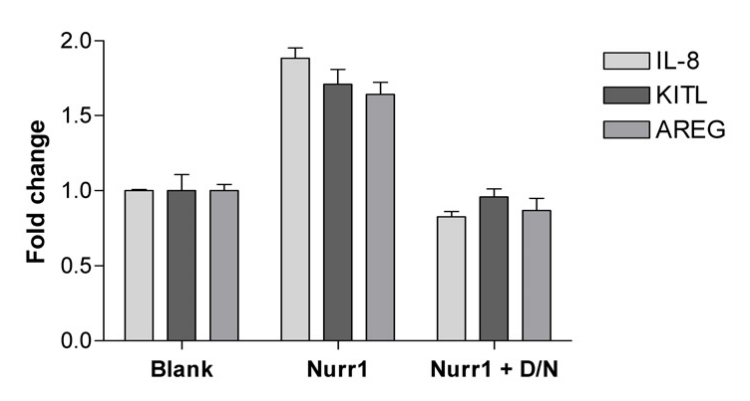
**Effect of dominant negative Nurr1 on Nurr1 induced inflammatory gene expression**. Demonstration that Nurr1 causes increased expression of IL-8, AREG and KITLG mRNA that can be attenuated with the coexpression of Nurr1-D/N in K4IM cells using Taqman Quantitative RT-PCR. Values expressed represent the mean fold change ± SEM, compared to the blank vector control and is derived from three experiments normalised in each case to GAPDH gene expression at 24 hr post transfection. Similar results were observed in a further two independent experiments.

In order to confirm the observations from the microarray analysis, K4IM cells were transfected with a Nurr1 expression construct and RNA harvested from cells after 16 hours. Quantitative RT-PCR analysis was then carried out with expression changes normalised to GAPDH. K4IM cells overexpressing Nurr1 showed increase expression of IL-8, KITLG and AREG transcripts compared to blank vector transfected cells confirming the observations from the microarray experiments (Figure 2). In addition consistent with the microarray data, the induced levels for each gene were returned to basal levels by co-transfection with the dominant negative Nurr1 construct (pcDNA3.1-Nurr1-DN) (Figure 2).

### Nurr1 dependent induction of IL-8 production in K4IM cells

**Figure 3:**
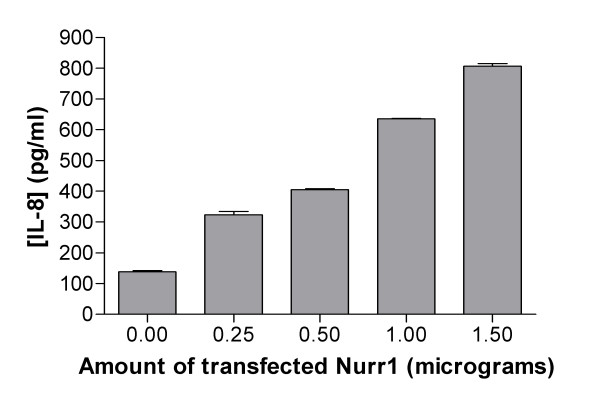
**Effects on increased expression of Nurr1 on IL-8 release**. 4 × 10^5 ^K4IM cells were transfected using the Amaxa Nucleofector with increasing amount of pCDNA3.1-Nurr1-WT, total amount of transfected DNA was 2 μg. Cell media was removed after 48 hour incubation and analysed for the amount of IL-8 present in the culture media using ELISA (R&D systems). Increased production of IL-8 protein secreted into the cell media was observed in a dose dependent manner with Nurr1 expression plasmid. Values expressed represent the mean concentration of IL-8 ± SEM.

To further confirm the functional consequences of elevated levels of Nurr1 we determined effects on IL-8 protein production using a sandwich ELISA on the media taken from Nurr1 transfected K4IM cells. The experiments were carried out using increasing concentrations of Nurr1 plasmid DNA (pcDNA3.1-Nurr1-WT) and a dose dependent increase in the amount of secreted IL-8 was observed (Figure 3). These results indicate that in K4IM cells elevated levels of Nurr1 leads directly to increased synthesis/secretion of the pro-inflammatory cytokine IL-8.

## Discussion

This study was designed to explore the potential link between Nurr1, pro-inflammatory signalling and the pathogenesis of rheumatoid arthritis using a synovial fibroblast derived cell line, K4IM as a model system. Transcript profiling via Affymetrix microarrays was used to examine the consequences of elevated Nurr1 levels in K4IM cells. Overexpression of Nurr1 in K4IM cells resulted in constitutive activity of the receptor as shown by the reporter assay and this activity could be suppressed by cotransfection with a dominant negative Nurr1 construct (Figure 1). Given that Nurr1 is an immediate early gene [19] and that transactivation activity using the reporter system is observed within hours of transfection (data not shown), we harvested RNA 16 hours following transfection to maximise the chance of identifying Nurr1 primary targets as opposed to downstream secondary effects. To have confidence in the Nurr1 dependent transcription of elevated genes we compared expression profiles between cells transfected with Nurr1 alone or with a mix of Nurr1 and dominant negative Nurr1. Using our stringent cutoffs, only three genes: IL-8, Amphiregulin and Kit ligand were upregulated more than 2-fold and subsequently returned to basal levels by the presence of the Nurr1 dominant negative, confirming the Nurr1 dependence. A list of probesets of 1.5-fold increase between Nurr1-WT and blank vector control transfections is provided as a supplementary table to show genes that may be either increasing or decreasing in their expression following Nurr1 transfection (see Additional file 1). This small number of differentially expressed genes may be representative of both the short time period of expression and the specificity of Nurr1 signalling. Importantly all three have recognized roles in inflammation. We examined in further detail the Nurr1-dependent induction of these genes in K4IM cells and confirmed the microarray observations using Taqman quantitative RT-PCR. Of these three genes, the most highly induced gene was the inflammatory cytokine, IL-8 and for this reason we further demonstrated using an IL-8 ELISA that Nurr1 specifically induces release of IL-8 protein into the culture media from K4IM cells (Figure 3). IL-8 has an established role in neutrophil recruitment via the CXCR1/2 receptors [29]

Amphiregulin and Kit ligand also have demonstrated roles in inflammation [30,31]. Transgenic mice expressing Amphiregulin under the control of keratin 14 promoter display early-onset synovial inflammation and severe skin pathology demonstrating a potential role for Amphiregulin in psoriasis and psoriatic arthritis [30]. Kit ligand, also known as Stem Cell Factor (SCF), has been shown to play a role in activation of mast cells. Administration of SCF into the airways of normal mice results in a dose dependent increase in airway hyperreactivity via mast cell activation demonstrating the role of SCF in the development of allergic airway inflammation and hyperreactivity [31]. In addition, activation of the Kit receptor by SCF leads to the phosphorylation of Akt which is necessary for IL-1-dependent NF-κB transactivation [32], Akt has been postulated to play a role in RA via its ability to regulate NF-κB and also promote resistance to apoptosis through a number of mechanisms [reviewed in [33]]. This role in cell cycle control remains consistent with other NR4 group members, in particular the Nur77 having an established role in TCR-mediated apoptosis of T hybridoma cells [34,35].

Nurr1 has previously been shown to act as a point of convergence for multiple inflammatory signals via CREB-1 and NFκB signalling [20]. Subsequent studies have shown Nurr1 and other NR4 family members to be involved in the inflammatory cascade of several stimuli, including TNFα-induced PAI-1 expression [36] and in LPS/TNFα stimulated macrophages [37]. TNFα plays a critical role in the stimulation of leukocyte recruitment and cytokine production and antibodies which act by blocking TNFα signalling have been shown to have clinically beneficial effects in RA patients [38]. Therefore we speculate that in addition to established NFκB signalling activating IL-8 expression [39], TNFα and other inflammation stimulators can act by regulating Nurr1 expression that in turn can regulate IL-8 expression either directly or indirectly. Ongoing work within our laboratory aims to address the exact mechanisms for Nurr1 activity on IL-8, Amphiregulin and Kit ligand gene expression.

In summary we have demonstrated using microarray analysis that elevated levels of Nurr1 leads to increased gene expression of IL-8, Amphiregulin and Kit ligand in the model cell line, K4IM. Moreover we have confirmed these Nurr1 dependent transcriptional changes using Taqman RT-PCR and demonstrated an increase in synthesis/secretion of IL-8 in cells transfected with Nurr1. We speculate that the elevated levels of Nurr1 observed in rheumatoid arthritis can potentially exacerbate the disease process in RA. Therefore, blocking the activation of Nurr1, or modifying the transactivation potential of Nurr1 through the use of rexinoids, methotrexate [40] or thiopurine analogues [41] represent a potential therapeutic option for rheumatoid arthritis and other inflammatory or allergic diseases.

## Competing interests

The author(s) declare that they have no competing interests.

## Authors' contributions

MRD designed the study, carried out the experiments, analysed the data, and drafted the manuscript. CJH carried out the plasmid construction work. SR and KT carried out the microarray analysis. MDJ, AEP and MRCN participated in study design and coordination as well as editing of the manuscript. All authors have read and approved the final manuscript.

## Supplementary Material

Additional file 1 **Differentially expressed genes following Nurr1 overexpression**. K4IM cells were transfected in triplicate using the Amaxa Nucleofector system for each of the 2 conditions: 1. 5 μg pcDNA3.1 blank vector (control); 2. 2.5 μg pcDNA3.1-Nurr1-WT (Nurr1 WT). Cells were cultured for 16 hours prior to RNA extraction. Genes were identified from the Affymetrix U133A chip showing significant change (>1.5 fold) between blank vector transfected synoviocytes and Nurr1 transfected K4IM cells (with a p-value of < 0.01). Fold changes in red are upregulated genes, those highlighted in green are downregulated genes, the previously identified genes: IL-8, AREG and KITLG are highlighted in boldface. Click here for file
