# Supplementary material for: Nurr1 dependent regulation of pro-inflammatory mediators in immortalised synovial fibroblasts
Source: J Inflamm (Lond). 2005 Nov 25;2:15. doi: 10.1186/1476-9255-2-15 (PMC1308852; doi:10.1186/1476-9255-2-15)
Supplement: Additional file 1 — Differentially expressed genes following Nurr1 overexpression. K4IM cells were transfected in triplicate using the Amaxa Nucleofector system for each of the 2 conditions: 1. 5 μg pcDNA3.1 blank vector (control); 2. 2.5 μg pcDNA3.1-Nurr1-WT (Nurr1 WT). Cells were cultured for 16 hours prior to RNA extraction. Genes were identified from the Affymetrix U133A chip showing significant change (>1.5 fold) between blank vector transfected synoviocytes and Nurr1 transfected K4IM cells (with a p-value of < 0.01). Fold changes in red are upregulated genes, those highlighted in green are downregulated genes, the previously identified genes: IL-8, AREG and KITLG are highlighted in boldface. [file 1476-9255-2-15-S1.doc]

| **Official HUGO Symbol** | Description | **RefSeqN Id** | **Fold change** | **p-value** |
| --- | --- | --- | --- | --- |
|  | hypothetical protein MGC35033 | NM_152319 | 12.18 | 0.00281 |
|  | HIV-1 rev binding protein 2 | NM_007043 | 6.49 | 0.00885 |
|  | Homo sapiens cDNA FLJ40901 fis, clone UTERU2003704 | AK098220 | 5.64 | 0.00472 |
| **IL8** | **interleukin 8** | **NM_000584** | **5.02** | **0.00047** |
|  | uridine-cytidine kinase 1 | NM_031432 | 4.98 | 0.00204 |
|  | Homo sapiens hypothetical protein MGC27277 complete cds. | BC042869 | 3.93 | 0.00341 |
|  | Human DNA sequence from clone RP5-1169J3 on chromosome 11p13 | HSJ1169J3 | 3.23 | 0.00089 |
| **AREG** | **amphiregulin (schwannoma-derived growth factor)** | **NM_001657** | **2.80** | **0.00037** |
|  | Homo sapiens amyloid beta (A4) precursor protein (protease nexin-II, Alzheimer disease) (APP) gene, complete cds | AY919674 | 2.35 | 0.00473 |
|  | **KIT ligand** | **NM_003994** | **2.23** | **0.00390** |
| EPOR | erythropoietin receptor | NM_000121 | 2.12 | 0.00410 |
|  | KIAA1414 protein | NM_019024 | 2.03 | 0.00466 |
| BIN1 | bridging integrator 1 | NM_139343 | 1.98 | 0.00093 |
| C1orf124 | chromosome 1 open reading frame 124 | NM_032018 | 1.95 | 0.00470 |
| PDHB | pyruvate dehydrogenase (lipoamide) beta | NM_000925 | 1.94 | 0.00270 |
|  | Homo sapiens cDNA FLJ42239 fis, clone TKIDN2002329 | AK124233 | 1.91 | 0.00342 |
| LRIG3 | leucine-rich repeats and immunoglobulin-like domains 3 | NM_153377 | 1.90 | 0.00481 |
|  | retinol dehydrogenase 10 | NM_172037 | 1.87 | 0.00397 |
| ATAD3A | ATPase family, AAA domain containing 3A | NM_018188 | 1.86 | 0.00658 |
|  | solute carrier family 17 (anion/sugar transporter), member 5 | NM_012434 | 1.85 | 0.00448 |
| TCEB3 | transcription elongation factor B (SIII), polypeptide 3 (110kDa, elongin A) | NM_003198 | 1.83 | 0.00456 |
| CCRN4L | CCR4 carbon catabolite repression 4-like (S. cerevisiae) | NM_012118 | 1.82 | 0.00139 |
| HFE | hemochromatosis | NM_139007 | 1.79 | 0.00472 |
| POGZ | pogo transposable element with ZNF domain | NM_015100 | 1.76 | 0.00705 |
| PCDH17 | protocadherin 17 | NM_014459 | 1.75 | 0.00221 |
| MGC5306 | hypothetical protein MGC5306 | NM_024116 | 1.73 | 0.00090 |
| PRSS15 | protease, serine, 15 | NM_004793 | 1.72 | 0.00188 |
| DSS1 | Deleted in split-hand/split-foot 1 region | NM_006304 | 1.71 | 0.00038 |
| NDUFA13 | Homo sapiens cell death-regulatory protein GRIM19 (GRIM19), mRNA | NM_015965 | 1.69 | 0.00246 |
| DDX49 | DEAD (Asp-Glu-Ala-Asp) box polypeptide 49 | NM_019070 | 1.69 | 0.00060 |
|  | nucleotide-binding oligomerization domains 27 | NM_032206 | 1.69 | 0.00312 |
| RBM5 | RNA binding motif protein 5 | NM_005778 | 1.68 | 0.00022 |
| C6orf18 | chromosome 6 open reading frame 18 | NM_019052 | 1.67 | 0.00192 |
| RG9MTD3 | RNA (guanine-9-) methyltransferase domain containing 3 | NM_144964 | 1.67 | 0.00941 |
| SMYD5 | Homo sapiens SMYD family member 5 (SMYD5), mRNA | NM_006062 | 1.65 | 0.00703 |
|  | homeo box A5 homeo box A3 | NM_019102 | 1.65 | 0.00373 |
|  | Homo sapiens hypothetical protein LOC348262, mRNA (cDNA clone IMAGE:4941803), partial cds. | BC063557 | 1.64 | 0.00751 |
|  | Homo sapiens chromosome 15 clone CTD-2295I7 map 15q15, WORKING DRAFT SEQUENCE, 3 ordered pieces. | AC022085 | 1.64 | 0.00664 |
| FKSG17 | FKSG17 | NR_002182 | 1.63 | 0.00284 |
| SNARK | likely ortholog of rat SNF1/AMP-activated protein kinase | NM_030952 | 1.63 | 0.00568 |
| C2orf30 | chromosome 2 open reading frame 30 | NM_015701 | 1.63 | 0.00455 |
| HSM802780 | Homo sapiens genomic DNA; cDNA DKFZp547D104 (from clone DKFZp547D104) | AL390168 | 1.62 | 0.00499 |
| SFRP1 | secreted frizzled-related protein 1 | NM_003012 | 1.62 | 0.00500 |
| PHIP | pleckstrin homology domain interacting protein | NM_017934 | 1.61 | 0.00531 |
| ZNF261 | zinc finger protein 261 | NM_005096 | 1.60 | 0.00264 |
| PCDH17 | protocadherin 17 | NM_003331 | 1.59 | 0.00506 |
| ZNF136 | zinc finger protein 136 (clone pHZ-20) | NM_003437 | 1.58 | 0.00408 |
| ITSN1 | intersectin 1 (SH3 domain protein) | NM_001001132 | 1.56 | 0.00858 |
| MTX3 | metaxin 3 | NM_001010891 | 1.56 | 0.00155 |
|  | hypothetical protein MGC45871 | NM_182705 | 1.56 | 0.00220 |
| MLL5 | myeloid/lymphoid or mixed-lineage leukemia 5 (trithorax homolog, Drosophila) | NM_182931 | 1.55 | 0.00406 |
|  | Homo sapiens chromosome 21 segment HS21C049 | HS21C049 | 1.53 | 0.00943 |
| MGC5139 | hypothetical protein MGC5139 | AL137327 | 1.53 | 0.00862 |
| FLJ90798 | hypothetical protein FLJ90798 | NM_153367 | 1.53 | 0.00604 |
| FLJ14639 | hypothetical protein FLJ14639 | NM_032815 | 1.52 | 0.00727 |
| HFE | hemochromatosis | NM_139007 | 1.52 | 0.00924 |
|  | Homo sapiens chromosome 3 clone RP11-96F15, complete sequence | AC097633 | 1.52 | 0.00021 |
| GTF2F2 | general transcription factor IIF, polypeptide 2, 30kDa | NM_004128 | 1.51 | 0.00418 |
